# Attentional network efficiency in elite biathletes and cross-country skiers

**DOI:** 10.3389/fspor.2026.1757734

**Published:** 2026-04-10

**Authors:** Irina S. Polikanova, Kirill S. Smirnov, Olga V. Sysoeva

**Affiliations:** 1 Faculty of Psychology, Moscow State University Named by Lomonosov, Moscow, Russia; 2 Laboratory of Convergent Studies of Cognitive Processes, FSBSI Federal Scientific Center for Psychological and Interdisciplinary Research, Moscow, Russia; 3 Faculty of Biology and Biotechnology, HSE University, Moscow, Russia; 4 Institute of Higher Nervous Activity and Neurophysiology, Russian Academy of Sciences, Moscow, Russia; 5 Center for Cognitive Sciences, Sirius University of Science and Technology, Sirius, Russia

**Keywords:** winter endurance sport, alerting, orienting, attention network test, elite athlete, sensitivity to temporal and spatial cues, optimal state of readiness

## Abstract

This study provides the first investigation of attentional network efficiency in elite winter endurance athletes, comparing biathletes and cross-country skiers. Utilizing the Attention Network Test (ANT), we assessed the orienting, alerting, and executive networks in 44 elite athletes (15 biathletes, 29 skiers) and 20 sedentary controls. The key findings reveal that the winter elite athletes together demonstrated a significantly lower alerting score compared to controls [13.1 ± 2.9 vs. 25.9 ± 5.2, Welsh's *t*_(63)_ = 2.134, *p* = 0.041, Cohen *d* = 0.592], interpreted as a neural signature of sustained readiness, where the nervous system is perpetually primed, diminishing the benefit of external warning cues. This capacity was manifested even more in biathletes than cross-country skiers and was also extended to spatiotemporal cues [combined score of alerting and orienting: 61.3 ± 9.4 vs. 90.2 ± 4.7, Welsh's *t*_(43)_ = 2.740, *p* = 0.012, Cohen *d* = 0.918]. This attenuated response to both temporal and spatial cues is posited as a trained “cognitive shield,” enabling biathletes to suppress automatic shifts of attention to spatial cues in parallel with sustained alerting state—a critical adaptation for maintaining precise marksmanship amidst the physiological noise and environmental distractions of shooting under high cardiovascular load. No group differences were found in executive attention networks and overall reaction time, underscoring the relevance of specific components of attention to these sports. The results demonstrate that the attentional architecture of elite athletes is not broadly enhanced but is precisely calibrated by their sport's unique demands, highlighting a remarkable degree of task-specific performance optimization under pressure.

## Introduction

Sport is an activity performed in unstable environments that requires highly flexible and effective attention, where the speed of reaction and decision-making can be crucial for success. Numerous studies have shown that athletes from various sports, from race car drivers to martial artists, exhibit faster reaction time than non-athletes (1–4, 43). This advantage even extends to domains like esports, suggesting a common foundation in superior perceptual-cognitive processing (5, 6). However, raw reaction speed alone is not the sole determinant of expertise. Research on attentional strategies shows that experts differ from novices in their visual search patterns, taking fewer but longer fixations (7, 8) and often employ specialized oculomotor strategies (9, 10, 42). Thus the quality of attentional control, rather than simple speed, is a key prerequisite for success in sport.

Attention is a complex cognitive function based on the interaction of various neural systems in the brain (44, 45). A powerful framework for investigating attention is Posner's model, which posits three fundamental attention networks: the executive (conflict) network, the orienting (selectivity) network, and the alerting (vigilance) network (11). The Attention Network Test (ANT) was developed to assess the efficiency of these three networks within a single behavioral paradigm (12) and examine the variations in the effectiveness of each of the three attention networks among individuals.

The application of the ANT in sports science indicates that athletes from different disciplines demonstrate unique patterns of attentional network efficiency, closely aligned with the demands of their sport. For instance, athletes from complex strategic sports such as soccer demonstrate difference in executive control and vigilance compared to static track and field athletes, aligning with the demands of tactical decision-making and response inhibition in a dynamically changing environment (13). Previous studies reported low conflict effect, indicating superior efficiency in executive network in resolving interference (14, 15, 46). For athletes in target-based precision sports like archery and shooting, research points to a more generalized decrease in reaction time across ANT conditions compared to non-athletes, suggesting a broad enhancement of processing speed that may underpin their specialized skill (16). For athletes in interceptive sports (table tennis, boxing, fencing, and free combat), in addition to superior conflict resolution ability, a higher overall RT was found compared to strategic sports (basketball, football, hockey, and rugby) and the control group (46). These findings collectively suggest that athletic expertise does not uniformly enhance all attentional networks but selectively tunes the systems most critical for a given sport's demands.

However, a significant gap remains. The ANT has not been previously applied to elite athletes in the closely related winter endurance sports of cross-country skiing and biathlon. These disciplines share the fundamental component of ski racing but are critically distinguished by the unique cognitive-motor challenge of shooting in biathlon. This task requires a rapid psychophysiological transition from high-intensity physical exertion to a state of precise marksmanship—a demand unparalleled in other sports. Shooting under fatigue exacerbates physiological tremor and impairs fine motor control (17, 18), placing a premium on exceptional cognitive regulation. To overcome this, elite biathletes develop highly specialized attentional and visuomotor strategies, such as stabilizing their gaze on the target to minimize movement (the “quiet eye” phenomenon), which is indicative of an optimized attention system for maintaining focus under stress (19, 20). This need to achieve a stable, trance-like state of mind amidst extreme physiological arousal defines the unique cognitive demands of biathlon (21). Consequently, we speculate that these specific demands have shaped a distinct attentional profile in biathletes, which can be delineated from their skiing counterparts through the framework of the Attention Network Test.

Our study aims to explore and compare the efficiency of all three attentional networks in elite biathletes and cross-country skiers, with a sedentary control group providing an opportunity to examine the effects that are specific to high athletic performance level. We hypothesize that elite athletes will exhibit enhanced efficiency in all three attentional networks—alerting, orienting, and executive control—compared to sedentary individuals. We also seek to identify the sport-specific attentional signatures associated with the unique demands of biathlon shooting vs. pure ski racing. We hypothesize that elite biathletes will be characterized by more sophisticated attentional control and orienting mechanisms compared to skiers and sedentary athletes, which will manifest as lower scores on the executive and higher scores in orienting components of the ANT.

## Materials and methods

### Participants

The study involved a total of sixty four Russian Caucasians (average age *M* = 27, SD = 4, range 18–34, ♂ = 44), who were divided into three groups: elite biathletes from the Russian national biathlon team (*n* = 15, ♂ = 11), elite skiers from the Russian national cross-country skiing team (*n* = 29, ♂ = 19), and sedentary controls (*n* = 20, ♂ = 14). While the sample was rather small, it consisted of the very high level athletes, and it was unfeasible to increase it. To examine our hypotheses we chose independent-samples Welch's *t*-tests, as this approach is more robust to unequal variances and sample sizes across groups, and directly addresses (1) the effect of expertise in winter endurance sport (comparison of biathletes + skiers with sedentary controls) and (2) the differences between biathletes and cross-country skiers attention networks. According to sensitivity analysis conducted using simulation code in MATLAB R2019b we would be able to detect with these analyses the effects of a large size (Cohen's *d* = 0.78 and 0.93, respectively) with alpha error probability of 0.05 and 80% power. This fact should be taken into account when interpreting the null effect.

The inclusion criteria for biathletes and skiers were the highest level of athletic skill, which was determined by their inclusion in national teams. The sedentary control group consisted of healthy volunteers from the same age group who had no experience in professional sports of any kind recruited from the University community.

The aim and procedures of the experiment were explained to the participants, and all of them provided written informed consent before the experimental session. The experimental protocol was approved by the local institutional committee of Moscow State University (Ethical Committee of the Psychological Science Department No. 09/2024, dated 15/01/2024) and Sirius University of Science and Technology (dated 03/09/2025) and was conducted in accordance with the Declaration of Helsinki.

### Procedure

The experimental procedure was similar to the variant of the ANT used by Fan, McCandliss, Fossella, Flombaum, Posner (22).

In this task, participants are required to press the left or right mouse button as soon as possible depending on the direction of the central arrow. Conflict arises from the surrounding flanker arrows, which can point in the same direction (congruent) or in the opposite direction (incongruent). Cues displayed before the target provide information about where or when the target will appear. Three scores were calculated, based on reaction time difference between conditions, reflecting each individual's performance for executive, orienting, and alerting components of attention. The accuracy was calculated as the relative value of correctly completed test trials.

**Figure 1:**
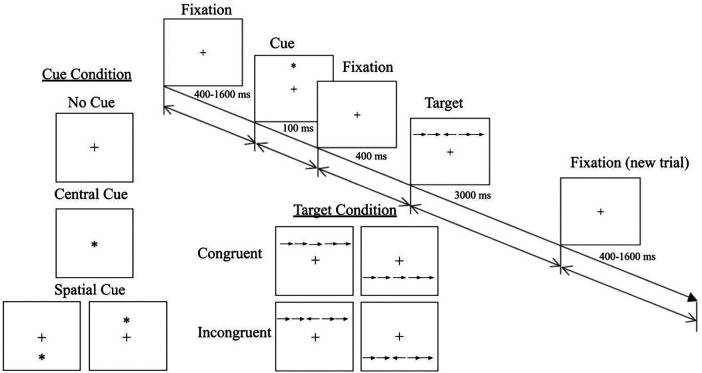
Schematic illustration of a trial in the ANT task with fixation, cue/no cue, target, and post-fixation time in ms.

The computerized ANT paradigm illustrated in Figure 1 consisted of 96 trials of five horizontally arranged black arrows (stimuli) that appeared either above or below a central fixation cross on the monitor against a gray background. The relative size of the arrow was about 0.6 degree and the spatial cues appeared about 1.0 degree above or below fixation cross. 32 stimuli were preceded by a central cue (alerting condition). Cues were presented before in the same position of appearance (above or below) of 32 stimuli (spatial orienting). The remaining 32 stimuli were without cue (no cue). 50% of the target stimuli were congruent. Targets were presented for 3,000 ms and response has to be done within this interval. Stimuli appear successively in a randomized order on the screen observed from a distance about 50 cm. The inter-stimulus interval (ISI) varied from 400 to 1,600 ms randomly.

The attention tests were conducted as a single session during the annual medical examination when the athletes had no competitions. They were held in the same bright room in the first half of the day, which helped minimize the influence of fatigue and stress, as the athletes were out of the training process. The humidity in the room was around 40%–50%. The temperature in the room ranged from 20 °C to 22 °C. The conditions were similar for the sedentary control group.

The efficiency of the three networks was assessed from the reaction time. The executive component of attention appears in “conflict” situations, i.e., when the target stimuli are surrounded by irrelevant and distracting stimuli. In our task the executive network score/conflict effect was calculated by subtracting the mean RT of all congruent flanking conditions from the mean RT of incongruent flanking conditions of the task (Executive = RT incongruent—RT congruent). Alerting component of attention is defined as a decrease of the response time for stimuli following central cues compared to no cue condition. Thus, the cueing does not point the place of where following stimuli appear, however, it creates a state of readiness for its rapid processing. Alerting component was calculated as a difference between reaction time with central cueing and those without it (Alerting = RT no cue—RT central cue). Orienting attention manifests itself when the target stimulus (a row of arrows) follows a spatial cue—when a signal indicates the location of the next stimulus. Orienting component was calculated as the difference between reaction time for the central cue and the response time for the spatial cue condition (Orienting = RT central cue—RT spatial cue) (22, 23).

### Statistical analysis

Statistical analysis was performed using Jamovi 2.4.1. Appropriate parametric statistical tests were conducted on meeting normality assumption and equal variance across the groups (Levene's Homogeneity of Variances Test).

While our main hypotheses were focused on attention networks, taking into account that measures of these networks are based on RT, we additionally examined all RT for each condition with one-way ANOVA separately for accuracy, no cue, central cue and spatial cuе, as well as congruent and incongruent conditions.

To assess the general effect of winter endurance sport expertise regardless of sport type we compared all athletes vs. sedentary controls using independent-samples Welch's *t*-tests separately for three components of attention: Executive, Orienting and Alerting. To examine the hypothesis regarding attention networks specifics between the group of athletes, we conducted similar analysis but contrasting biathletes vs. cross-country skiers.

## Results

For each participant, mean correct RT after eliminating extreme values (less than 200 ms and more than 1,200 ms: 2% of the total) and mean error rate were computed and subjected to analyses (14, 24). The RT and attention networks scores were expressed in ms, accuracy was presented in %. The normality and the homogeneity assumptions of the data were not violated. Accuracy, RT for each group and condition are presented in Table 1 as mean and SD with results of the ANOVA analysis. No between group differences were found.

**Table 1:** Accuracy and RTs for each condition and group with results of the ANOVA analysis.

Group/Statistic	Accuracy	Mean RTs (SE)					
		Overall	Flanker type		Cue type		
			Congruent	Incongruent	No	Central	Spatial
Biathletes (*n* = 15)	97.5 (0.73)	541 (13)	492 (13)	594 (15)	565 (15)	560 (14)	504 (13)
Skiers (*n* = 29)	97.7 (0.37)	546 (11)	494 (9)	597 (15)	584 (12)	567 (13)	494 (12)
Sedentary controls (*n* = 20)	96.5 (094)	564 (20)	516 (18)	617 (23)	604 (22)	578 (21)	514 (18)
*F*	0.662	0.459	0.717	0.341	1.135	0.267	0.474
*p*	0.524	0.636	0.496	0.713	0.333	0.767	0.626
Partial *η*²	0.031	0.019	0.032	0.014	0.037	0.009	0.018

RT, reaction time, expressed in ms; SE, standard error.

**Figure 2:**
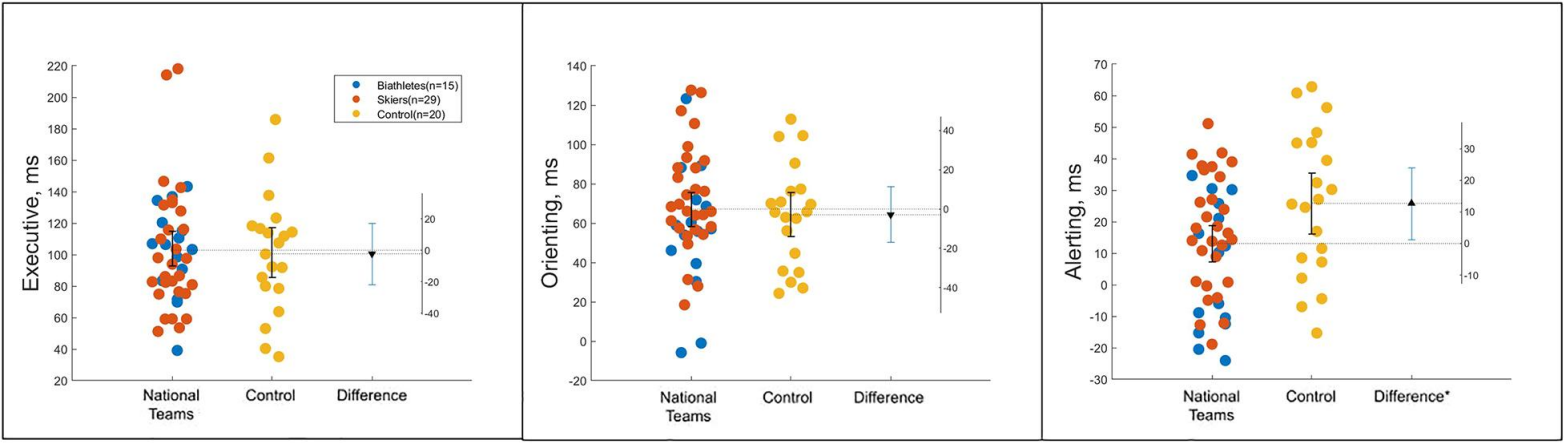
Estimation plots of executive, orienting and alerting components of the ANT test for athletes from national teams (combined biathletes and cross-country skiers) and for sedentary controls. Only difference in alerting scores reached significance at the level of *p* < 0.05. Each dot represents a participant.

Table 2 contains attention network scores with the results of one-way ANOVA for the executive, alerting, and orienting components of the ANT compared across the two groups (elite athletes and sedentary controls), which was done to examine our first hypothesis about the non-specific effect of expertise in winter endurance sport on the attention networks. Only the alerting effect significantly differentiated professional athletes from sedentary controls. Figure 2 represents the between group difference for each component of attention.

**Table 2:** The attention networks scores (mean, ms and SE-standard error) for national teams athletes (combined biathletes and skiers) and control groups with the results of Welch's *t*-test.

Group/Statistic	Executive		Orienting		Alerting	
	Mean	SE	Mean	SE	Mean	SE
National teams (biathletes + skiers) (*n* = 44)	102.8	5.6	67.2	4.52	13.1	2.97
Sedentary controls (*n* = 20)	100.58	8.39	64.3	5.84	25.86	5.18
Welch's *t* test	−0.225		−0.398		**2.134**	
*p*	0.823		0.693		**0.041** *	
Cohen *d*	0.061		−0.104		0.592	

RT, reaction time, expressed in ms; SE, standard error.

Significant differences are marked in bold.

* *p* ≤ 0.05.

**Figure 3:**
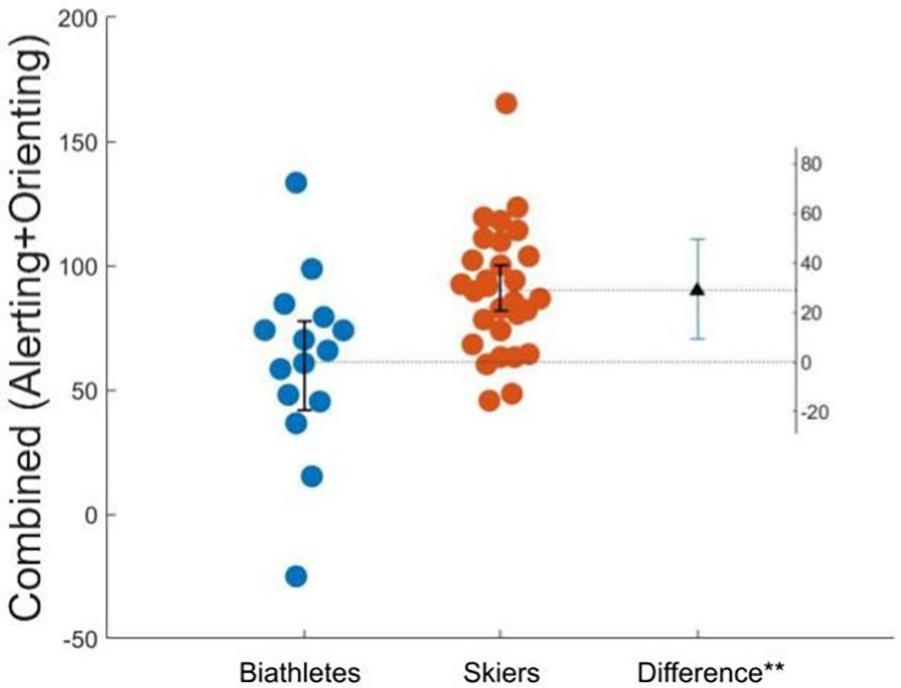
Estimation plot of combined scores of alerting and orienting attention networks for elite biathletes and cross-country skiers. The difference is significant with *p* < 0.05. Each dot represents a participant.

Table 3 represents the results of the statistical analysis for the executive, alerting, and orienting components of the ANT compared between biathletes and skiers, which was done to examine the specific difference in attention networks between two types of winter endurance sport. As components of alerting and orienting were both smaller in biathletes, but did not reach significance, we decided to combine those measures to assess a general sensitivity to external cues. Combined scores were calculated as Alerting + Orienting. This combined measure appeared to be significantly lower in biathletes compared to skiers as also represented in Table 3 as well as on Figure 3.

**Table 3:** The attention networks scores (mean, ms and SE-standard error) for biathletes and skiers with the results of Welch's *t*-test.

Group/Statistic	Executive		Orienting		Alerting		Combined Orienting and Alerting	
	Mean	SE	Mean	SE	Mean	SE	Mean	SE
Biathletes (*n* = 15)	102.05	7.24	55.8	8.5	5.56	5.26	61.3	9.39
Skiers (n = 29)	103.26	7.72	73.1	5.01	17.04	3.43	90.2	4.72
Welch's *t* test	−0.114		−1.755		−1.827		**−2.740**	
*p*	0.910		0.092		0.079		**0.012** *	
Cohen *d*	−0.034		−0.576		−0.590		−0.918	

RT, reaction time, expressed in ms; SE, standard error.

Significant differences are marked in bold.

* *p* ≤ 0.05.

## Discussion

The RT, accuracy and attention network scores, obtained in our study, generally corresponds with previous literature on ANT. The mean values reported in most studies ranged within the following values: 450–750 ms for RT, 85%–99% for accuracy, 7–60 ms for alerting, 25–65 ms for orienting, and 50–110 for executive scores (13, 14, 16, 22, 25, 46). The rather wide range might be attributed to different versions of the task (e.g., alerting tones or visual cues, different timing of the stimuli) and different samples.

While being within the context of previous research, our study provides the new data about attentional network profiles in elite biathletes and cross-country skiers. Both groups of athletes demonstrated a significantly reduced alerting effect compared to sedentary controls, providing first evidence for low sensitivity to temporal cue in experts of winter endurance sport. However, while we expected to find a distinct sport-specific effect within these types of sports, in particular, a more efficient executive and orienting attention networks in biathletes, our hypothesis was not supported. Moreover, the only effect that was close to significance was for the orienting network and in the opposite direction: orientation networks scores were lower in biathletes, indicating their less sensitivity to spatial cues. At the same time all negative findings of our study should be taken with caution due to the small sample size that was sufficient only for the effect of large size. Nevertheless, below we discuss the obtained results in more detail.

### Low benefits of temporal cue in elite athletes: optimal state of readiness

According to Posner (26) the high alerting scores may be explained by several factors, such as inability to maintain the alerting state without a cue and from more efficient reaction to the alerting cue. The first factor is linked to low tonic alertness, while the second to a high phasic alertness. Tonic alertness is a long-term, stable state of vigilance, while phasic alertness refers to short-term, transient increases in alertness in response to specific cues (26). We propose that the constant high-stakes environment of elite sport necessitates a constant state of preparedness. For these athletes, the nervous system may be “always primed,” diminishing the additional benefit of a phasic cue. This finding is clarified by the modern understanding of the alerting network as an emergent property of dynamic shifts between core intrinsic networks. Effective phasic alerting typically requires the rapid recruitment of the dorsal attention (DAN) and midcingulo-insular (M-CIN) networks, coupled with the reciprocal deactivation of the fronto-parietal control network and the default mode network (DMN) (27). The lower alerting score in elite athletes suggests their systems are already operating at an optimal baseline, potentially characterized by a more stable and resistant DMN and a more efficient fronto-parietal system. This optimized baseline state would necessitate a smaller dynamic shift for a phasic cue, resulting in a diminished behavioral alerting effect. Thus, the lower score is not a deficit but a signature of a neural system that has achieved a state of high readiness where external prompts are superfluous.

Our interpretation finds strong support in the work of Rahimi et al. (13), who also observed a reduced alerting effect in athletes from strategic sports (soccer players) (alerting component—7) compared to those in static sports (track and field) (alerting component—14).

This convergence of findings across disparate disciplines—from soccer to skiing and biathlon—suggests that a reduced reliance on phasic warning cues may be a common feature of expertise in sports that require constant anticipation and resolution of temporal uncertainty. In soccer, this involves anticipating opponents' moves and the ball's trajectory; in cross-country skiing, it entails optimal pacing and reacting to competitors; in biathlon, it is crucial for regulating breathing and heart rate in preparation for shooting. The ability to maintain an internally generated, tonic state of readiness, thereby minimizing dependence on external prompts, appears to be a key adaptation that transcends a specific sport's mechanics. This aligns with research outside the ANT paradigm that reported athletes’ superiority only for 500 ms foreperiod with no difference between performance with 500 and 1,500 ms foreperiods in tennis players, suggesting constant readiness (28). Thus, the lower alerting score in the athletes of our study can be viewed as the behavioral signature of this optimized system for resolving temporal uncertainty—a system so adept at maintaining preparedness that the utility of a simple warning signal is minimized.

This interpretation elegantly resolves the apparent paradox of a “reduced” alerting effect being a marker of expertise. It is not that athletes are worse at becoming alert; rather, their system is already operating at an optimal baseline, requiring a smaller dynamic shift to achieve a state of high readiness. The lower alerting score is thus the behavioral signature of a neural system that has reached an efficient equilibrium, maintaining preparedness without needing strong external prompts. This optimized state likely reflects a refined balance within the norepinephrine system, which modulates the cortical excitability underlying tonic vigilance, allowing for a state of readiness that is both sustained and metabolically economical (29, 47).

### Less sensitivity of biathletes to external cues: the biathlete's shield

Our second aim of the study was to reveal sport-specific tuning of attention networks. In particular, we hypothesized that executive and orienting networks would operate more efficiently in biathletes than cross-country skiers. Our results clearly did not support it. Moreover, orienting and alerting scores even have a tendency to be lower for biathletes than cross-country skiers, indicating less sensitivity to both temporal and spatial cues in biathletes, compared to cross-country skiers. The combined measure of the sensitivity to both temporal and spatial cues clearly differentiated biathletes from skiers. The decision to combine alerting and orienting scores is supported by neuroimaging evidence demonstrating overlapping neural substrates for these networks. Wang et al. (30) identified shared involvement of the temporo-parietal junction in both alerting and orienting. Extending these findings, Markett et al. (27) used intrinsic connectivity analysis to show that all attention networks converge in the dorsal fronto-parietal and midcingulo-insular networks, indicating that attention is supported by a distributed system with integrated hubs rather than completely independent networks. This shared neurobiology provides a theoretical rationale for integrative assessment of cue-related attentional functions in the context of athletic expertise. Below we discuss this unexpected finding.

Less sensitivity to temporal cues was discussed in previous session and here we can indicate a tendency to even larger degree of readiness in biathletes compared to pure race skiers. At the same time, cross-country skiers did not demonstrate any sign of decreased sensitivity to spatial cue, thus, making this characteristic specific to biathletes. The orienting network, which facilitates rapid, often automatic, shifts of attention to salient spatial cues (11), appears to be attenuated in this group. This ability might be a trained and essential component of their shooting expertise. The orienting network prioritizes sensory inputs through both exogenous (stimulus-driven) and endogenous (intentional) attention, suggesting a more integrated view of attentional processing rather than a strict dichotomy between the two (31). During the precision task of shooting—executed under high cardiovascular load, physiological tremor, and unpredictable environmental conditions like shifting wind (17, 19)—an automatic orienting response to any peripheral movement or cue would be catastrophic for performance.

This adaptation can be understood through multiple theoretical lenses. At its core, it reflects a powerful dominance of the goal-directed (top-down) dorsal attention network over the stimulus-driven (bottom-up) ventral attention network (29, 32). The intensive training of biathletes, involving thousands of shots under deliberately varied and distracting conditions (21), effectively teaches the brain to gate out irrelevant spatial information to protect a critical motor program. This is the cognitive substrate of the “quiet eye” (QE)—the prolonged final fixation on the target that is a hallmark of expert shooting (19, 20, 33). The attenuated orienting score in the lab is the direct behavioral counterpart to this stable QE on the shooting range. The reduced orienting score demonstrates their ability to resist the automatic pull of spatial cues in the ANT, a task that directly conflicts with the narrow-external focus required for shooting. This can be seen as a form of strategic modulation, where athletes suppress automatic orienting in favor of sustained concentration.

This profile stands in stark contrast to findings in other precision sports, such as archery and shooting, where elite athletes did not show either reduced orienting or alerting effect and scores for these attention networks are even increased with mindfulness training (16). In particular in these target-based precision sports the orienting and alerting components were almost indistinguishable from the control group (orienting: shooting—63, archery—56, controls—68; alerting: shooting—25, archery—29, controls—25). This discrepancy is likely the key to understanding the unique cognitive demands of biathlon. Unlike static marksmanship, biathlon requires executing precision under high physical endurance. The critical factor is not just focus, but “noise resistance”—the ability to maintain that focus amidst the physiological noise of extreme exertion. The suppressed orienting network we observed appears to be a specific adaptation to this challenge, a specialized cognitive filter that is less necessary for shooters in a state of physiological rest. At the same time we cannot disregard the potential cross-culture difference in ANT as well as training strategies of athletes, thus further studies are needed to more deeply explore this issue.

In conclusion, the lower sensitivity to both temporal and spatial cues in biathletes is a testament to targeted neural plasticity. It represents an optimized attentional configuration: a system strategically tuned to prioritize stability over exploration, and to protect its focus through a pre-emptively suppressed orienting response, making it uniquely suited to the extreme demands of the sport. This finding underscores that “enhancement” in attention is not a uniform concept; for a biathlete, it means cultivating a powerful resistance to any external cues that is beneficial in other athletic contexts.

### The null finding: executive control and general processing speed

The absence of group differences in executive control is also highly informative. It suggests that the cognitive expertise of these winter sport endurance athletes is not manifested as a generalized enhancement in resolving internal conflict, as might be seen in open-skill, strategic sports like soccer and tennis (13, 15, 25, 34). In particular, the executive component in soccer players was smaller (96 ms) as compared to track and field athletes (109 ms) (13); in college table tennis players (54 ms) compared to non-athletes (69 ms) (25); and for athletes in interceptive sports (table tennis, boxing, fencing, and free combat, 67 ms) compared to strategic sports (basketball, football, hockey, and rugby, 94 ms) and the control group (106 ms) (46). At the same time for shooting and archery the executive component was similar to controls being 87, 89 and 92 ms, respectively (16). To sum up, resolving a flanker arrow conflict seems to be not among key professional abilities neither for winter endurance sport nor for shooting and archery and track and field athletes.

Similarly, the lack of a general reaction time advantage underscores the specificity of the observed attentional effects. While elite athletes are often reported to have faster simple reaction times in sport-specific contexts, our results demonstrate that this general sensorimotor advantage does not necessarily extend to a non-specialized laboratory task like the ANT. This key finding suggests that the cognitive expertise of these athletes is not manifested as a generalized speed advantage in basic perceptual-motor processing. Instead, their superiority lies in a more efficient and optimized regulation of attentional resources—specifically, a reduced dependency on external cues for alerting. This pattern indicates a highly trained, resource-efficient system where optimal level of readiness is the default state, eliminating the need for additional preparatory cues and providing a neural buffer against distractions.

### Theoretical integration: automaticity and focus

Our findings dovetail with established theories of expert performance. The reduced reliance on external cues supports the concept of skill automaticity, where highly practiced actions become proceduralized and require less conscious, cue-dependent control (35). This automaticity is a key defense against “choking under pressure,” (36–38), a phenomenon where explicit monitoring disrupts automated skills (39). For a biathlete, whose success depends on performing a precise motor skill under extreme pressure and fatigue, a brain that automatically resists distractions and maintains focus is a brain that is less likely to choke. Furthermore, the pattern we observed is entirely consistent with the advantages of an external focus of attention (40, 41). By focusing on the movement's effect (e.g., the rifle's alignment with the target) rather than the movement itself (e.g., their finger on the trigger), athletes achieve more robust and efficient performance. The biathletes’ suppressed response to external cues may be the neural manifestation of a deeply ingrained external focus, so firmly locked on the task goal that it is immune to the pulling effect of arbitrary spatial cues.

### Limitations and future directions

This study has several limitations. First, the sample size is relatively small, consisting of 64 participants, of whom only 15 were biathletes. Recruiting more biathletes was unfeasible due to our focus on high-level athletes of national team caliber. Consequently, our sensitivity analysis indicated that we could only reliably detect effects of a large size (*d* > 0.78), which limits the interpretation of null findings. Future research should aim to expand the sample size and potentially examine the effect of the level of expertise. Including athletes of varying qualification levels would contribute to a more nuanced analysis of the development of attention specialization.

The issue of effect size is particularly relevant to our findings. For the biathlete vs. skier comparison, the observed effect for the combined scores of alerting and orienting networks was large (*d* = 0.92) and statistically robust. However, when alerting and orienting networks were analyzed separately, the medium-sized effects (*d* = 0.57–0.59) did not reach statistical significance. Additionally, the difference in alerting for the athlete vs. control comparison were also of medium size (*d* = 0.59). Therefore, these findings should be interpreted with caution and require replication in independent research with larger samples to confirm the observed trends.

Second, the laboratory setting of the Attention Network Test (ANT), while methodologically standardized, lacks the ecological validity of on-snow performance, which may limit the generalizability of our findings to real-world competitive environments. The athletes in sports such as biathlon and cross-country skiing compete in dynamic real-world environments that involve factors such as wind, physical fatigue, and fluctuating natural conditions. Therefore, any observed differences in the orienting and alerting attention networks may reflect laboratory-specific effects rather than the athletes’ true attentional capacities during actual competition. And on the contrary, lack of the effect on the executive network might be a limitation of the laboratory setting as well as low sample size of the study. Incorporating field-based simulations or more ecologically valid training environments, are important directions for the future research to ensure that laboratory findings accurately reflect the athletes’ real-world performance.

Third, our experimental paradigm utilized only valid spatial cues. While this design effectively measures the typical benefit of orienting, it precluded the examination of responses to invalid cues. According to our hypothesis of a trained “cognitive shield,” the ability to minimize performance costs from invalid or distracting cues is precisely the skill we would predict to be enhanced in elite biathletes. Therefore, a crucial direction for future research would be to employ a modified ANT that incorporates both valid and invalid spatial cues. Such a design would allow researchers to dissect whether the attenuated orienting effect in biathletes stems from a generalized dampening of the orienting response, or a more sophisticated and efficient ability to specifically suppress distraction from invalid cues while still potentially benefiting from highly reliable ones. This would provide a more nuanced understanding of the highly refined attentional control developed through biathlon training.

Future research will also benefit from incorporating physiological measures (e.g., EEG, pupillometry) to directly probe the neural correlates of tonic alertness and inhibitory control during task performance.

## Conclusion

Our hypothesis that elite biathletes would be characterized by more sophisticated attentional control mechanisms compared to skiers and sedentary participants—manifesting as faster reactions to incongruent stimuli of the flanker type and spatial cue type, as well as lower scores on the executive and higher scores for orienting components of the ANT Test—was not confirmed. However, an unexpected finding was that both groups of athletes demonstrated significantly reduced benefits of temporal cues compared to sedentary controls, which was most pronounced and even extended to the spatial cues in biathletes. Thus, the attentional architectures of elite biathletes and cross-country skiers are not broadly enhanced but are precisely and differentially calibrated by their unique performance demands. Both groups exhibit a neural optimization for sustained readiness and superior temporal preparation, reflected in a reduced phasic alerting effect. Biathletes alone develop an additional, critical adaptation: a suppressed orienting response that acts as a cognitive shield against environmental distractions, enabling precise marksmanship under stress. These findings highlight the extraordinary capacity of the human brain for task-specific plasticity, transforming fundamental attention networks into a finely tuned instrument for elite athletic performance.

## Data Availability

The raw data supporting the conclusions of this article will be made available by the authors, without undue reservation.
